# Optogenetic control of morphogenesis goes 3D

**DOI:** 10.15252/embj.2018100961

**Published:** 2018-11-22

**Authors:** Barry James Thompson

**Affiliations:** ^1^ Cancer Research UK London UK

**Keywords:** Cell Adhesion, Polarity & Cytoskeleton, Development & Differentiation

## Abstract

The generation of form in living embryos, a process termed “morphogenesis” from the Greek word μορφογένεση, is one of the most fascinating unsolved problems in biology. In embryonic epithelia, most attention has been paid to events occurring at the apical surface of epithelia, particularly the regulation of actomyosin contractility during morphogenetic change. In a new report, De Renzis and colleagues demonstrate a key role for regulated actomyosin contractility at the basal surface of the epithelium during formation of the first epithelial fold in *Drosophila* (the “ventral furrow”) (Krueger *et al*, 2018).

Epithelia are the most ancient type of animal tissue, being present in all animals, including those derived from the very base of the metazoan evolutionary tree. Epithelia are also typically the first type of tissue to arise in animal development, appearing during or immediately after the blastocyst stage of embryogenesis. In many species, establishment of the first epithelium is followed by formation of a single epithelial fold, namely the “primitive streak” in vertebrates or the “ventral furrow” in *Drosophila*. Genetic screens in *Drosophila* identified the *twist* and *snail* genes as being required for epithelial folding and the subsequent process of epithelial‐to‐mesenchymal transition (EMT) that produces mesodermal tissues inside the protective outer epithelium (Simpson, 1983; Nusslein‐Volhard *et al*, 1984).

The *Drosophila* ventral furrow has since become an excellent model system for understanding how epithelial folding can occur, with numerous studies demonstrating the importance of apical actomyosin‐driven constriction in driving indentation of the epithelium (Leptin & Grunewald, 1990; Sweeton *et al*, 1991; Dawes‐Hoang *et al*, 2005; Martin *et al*, 2009). Thanks to these impressive studies, it is often thought that the problem of ventral furrow formation is now solved. However, computer simulations indicate that apical constriction is not actually sufficient to explain the complete invagination of the epithelium. Instead, the models suggest that apical constriction must be accompanied by basal relaxation to achieve the pyramidal cell shape observed in folds. Just such a disassembly of basal contractile actomyosin has been visualised during ventral furrow formation (Sweeton *et al*, 1991; Dawes‐Hoang *et al*, 2005). However, whether basal relaxation is truly necessary for folding remained unclear, as some models point to basal relaxation as a passive consequence of apical constriction (Polyakov *et al*, 2014).

In a new study, De Renzis and colleagues deploy optogenetic control of actomyosin contractility to prevent basal relaxation and consequently disrupt ventral furrow formation. The findings unequivocally demonstrate the essential role of basal relaxation in allowing pyramidal cell shape changes necessary for full folding of the epithelium. The authors voice their support for the model that apical constriction, which recruits myosin‐II, may ultimately cause basal relaxation through passive depletion of myosin‐II. Nevertheless, it will be interesting to determine whether specific molecular mechanisms exist to antagonise basal myosin‐II recruitment and thus promote basal relaxation during ventral furrow formation.

The findings in the *Drosophila* embryo are likely to be relevant to epithelial morphogenesis in other developmental stages and other species. The basal surfaces of many different epithelia experience dynamic changes in locally generated actomyosin contractile forces (He *et al*, 2010; Sherrard *et al*, 2010; Sun *et al*, 2017; Huebner & Wallingford, 2018) as well as in the constraining force of the basal extracellular matrix (Haigo & Bilder, 2011; Diaz‐de‐la‐Loza *et al*, 2018). Thus, a full understanding of epithelial morphogenesis will require moving from 2D analysis of the apical surface towards 3D analysis of tissue mechanics at both the apical and basal surface of epithelia.
